# Racial and ethnic characteristics and cancer-specific survival in Primary Malignant Cardiac Tumors

**DOI:** 10.3389/fcvm.2022.961160

**Published:** 2022-08-25

**Authors:** Mark M. Aloysius, Sanskriti Shrivastava, Chaitanya Rojulpote, Raza Naseer, Hamza Hanif, Milos Babic, Kenneth Gentilezza, Pranjal K. Boruah, Samir Pancholy

**Affiliations:** ^1^Department of Internal Medicine, The Wright Center for Graduate Medical Education, Scranton, PA, United States; ^2^Department of Physical Medicine and Rehabilitation, The Wright Center for Graduate Medical Education, Scranton, PA, United States; ^3^Department of Cardiovascular Diseases, The Wright Center for Graduate Medical Education, Scranton, PA, United States

**Keywords:** risk factor, disparities, epidemiology, outcomes, race, ethnicity, Primary Malignant Cardiac Tumors

## Abstract

**Background:**

There is limited insight into the epidemiological characteristics and effect of race and ethnicity on Primary Malignant Cardiac Tumors (PMCTs).

**Objectives:**

Comparison of clinical characteristics and cancer-specific survival outcomes of major races in the United States from the Surveillance, Epidemiology and End-Result (SEER) registry.

**Methods:**

ICD-O-3 codes were used to identify PMCTs for the years 1975 to 2015. Three major races were identified—“White”, “Black”, and “Asian/Pacific Islander”. Cancer-specific survival outcomes were compared using Kaplan-Meier analysis across and amongst races, based on tumor histology. A subgroup analysis of cancer-specific survival was performed between “Hispanics” and “non-Hispanics.”

**Results:**

Seven hundred and twenty patients were identified−47% females and 79% White, mean age at diagnosis (47 ± 20 years). Black patients were significantly younger (39 ± 18 years) and presented more commonly with angiosarcomas (53%). Non-angiogenic sarcomas and lymphomas were the most common tumors in the White (38%) and Asian/Pacific Islander (34%) cohorts. For a median follow-up period of 50 (IQR3-86) months, cancer-specific survival (mean ± SD, in months) was worse in Blacks (9 ± 3) as compared to Whites (15 ± 1) and Asian/Pacific Islander (14 ± 1) (*p-*value; Black vs. White <0.001; Black vs. Asian/Pacific Islanders = 0.017, White vs. Asian/Pacific Islanders = 0.3). Subgroup analysis with 116 (16%) Hispanics (40% females; mean age of 40 ± 20 years) showed a longer mean cancer-specific survival of 16.9 ± 2.4 months as compared to 13.6 ± 1.1 months in non-Hispanics (*p* = 0.011).

**Conclusion:**

Black and non-Hispanic patients have poorer cancer-specific survival in PMCTs.

## Introduction

Primary malignant cardiac tumors (PMCTs) are rare, representing only 0.008% of all cancers reported in the United States (1). Affected patients tend to remain asymptomatic early in the disease course with aggressive clinical presentation in later stages with complaints of chest pain, dyspnea, syncope, and hemoptysis (2, 3). Seventy percent (70%) patients present with congestive heart failure but at times, sudden cardiac death is the only manifestation of these tumors (4). These tumors are histologically diverse with limited treatment options and most patients face a dismal prognosis. Current literature on PMCTs is based on single-center studies and autopsy evaluations with small sample sizes leading to limited insight into the tumors' sociodemographic behavior (3, 5).

The National Cancer Institute's (NCI) initiative SEER (Surveillance, Epidemiology, and End-Result) obtains data from 18 cancer registries and is the largest database of cancers in the United States. There is an observed upward trend in reporting of PMCTs over the years in SEER, especially with recent advancements in the field of cardiac imaging and has allowed researchers to draw important conclusions related to sexual and histologic differences (6, 7).

Race and ethnicity are considered strong risk factors in the incidence and outcomes of various malignancies and interplay of genetic factors and socioeconomic determinants of health have been implicated (8, 9). At this time, very limited knowledge exists on the role of race and ethnicity in the presentation, survival, and prognosis of PMCTs. This study aims to use the population-based SEER database to demonstrate—(1) Differences in characteristics and cancer-specific survival outcomes of PMCTs between different races in the United States, (2) Differences in cancer-specific survival outcomes for diverse histologic types of PMCTs within each race, and (3) Identify ethnic differences in characteristics and cancer-specific survival outcomes of PMCTs in Hispanics as compared to non-Hispanics in the United States.

## Materials and methods

An exemption was obtained from the Wright Center for Graduate Medical Education Institutional Review Board in Scranton, PA (IRB#1698777-1). A retrospective cohort study design was adopted. Deidentified data from the (NCI's SEER database, a cancer registry from a total of 17 sites in the United States was collected utilizing the SEER Stat (v 8.3.9^®^ NCI). SEER database is supported by the Surveillance Research Program (SRP) in the Division of Cancer Control and Population Sciences (DCCPS). Author M.M.A had full access to the study data and takes full responsibility for its integrity and data analysis. ICD-O-3 code “C 38.0” (Heart) was used for initial extraction of patients between the years 1975 to 2015 which revealed 826 malignant cardiac tumors. Inclusion criteria consisted of patients older than 18 years with the diagnosis of PMCTs established on tumor histology. Patients with >1 primary tumor were excluded. This approach is consistent with previous studies utilizing the SEER database for epidemiological research (6). Out of the 727 patients meeting inclusion and exclusion criteria, 6 Alaskan Natives and 1 patient with an unidentified racial description were excluded from the statistical analysis. Demographic and clinical characteristics were obtained for 720 patients consisting of 3 major races—“White”, “Black”, and “Asian/Pacific Islander”. Four broad histologic types-angiosarcomas—non-angiogenic sarcomas, lymphomas, and mesotheliomas were reported. SEER staging was used to report tumor stage due to relative uniformity over the years. Finally, a subgroup analysis was performed by a reclassification of the cohort into “Hispanics” and “non-Hispanics” as predefined in the SEER database to determine the role of ethnicity in the characteristics and outcomes of PMCTs.

### Statistical analysis

Statistical analysis was performed using SPSS v 28^®^ IBM. Demographic and clinical characteristics were represented using mean and standard deviation if normally distributed or median with interquartile range, if non-parametric. The difference between characteristics was analyzed using an independent sample *t*-test or Mann–Whitney-*U*-test for continuous variables and a chi-square test for categorical variables. Pairwise comparisons for cancer-specific survival were performed using the Mantel-Cox (log-rank) test and Kaplan-Meier survival curves were constructed. A *p*-value of <0.05 was considered statistically significant for all outcomes.

## Results

In 720 patients with PMCTs, 47% of patients were female with the mean age at diagnosis of 47 ± 20 years. Five hundred and seventy-two (79%) patients were White, 75 (11%) were Black and 73 (10%) were Asian/ Pacific Islanders. Details of the complete PMCT cohort are illustrated in Table 1. No gender differences were noted in PMCT across races. Non-angiogenic sarcomas were more commonly seen among Whites (38%) followed by Asian/Pacific Islanders (27%) and Blacks (17%) (*p* = 0.0003). Angiosarcomas were the predominant histologic type in Blacks (53%), followed by Asian/Pacific Islanders (33%) and Whites (25%) (*p* < 0.0001). On the other hand, lymphomas were more common in Asian/Pacific Islanders (34%) as compared to White (29%) and Black (20%) populations. Mesotheliomas were the least common type of tumor across all races representing 9% Blacks, 7% Whites, and 5% Asian/Pacific Islanders with no statistically significant difference in presentation (*p* = 0.651). Baseline characteristics for each race is illustrated in Table 2. In-depth histological description of all tumors has been provided in the Supplementary material.

**Table 1:** Characteristics of all Primary Malignant Cardiac Tumors (PMCTs).

**Characteristics**	**All PCMTs (*n* = 720)**
Age at diagnosis, y ± SD	47 ± 20
Female *n* (%)	337 (47)
Hispanics, *n* (%)	116 (16)
Insured, *n* (%)	327 (45)
**Histology** ***n*** **(%)**	
Angiosarcoma	208 (29)
Non-angiogenic sarcoma	253 (35)
Lymphoma	208 (29)
Mesothelioma	51 (7)
**SEER staging** ***n*** **(%)**	
Localized	136 (19)
Regional	129 (18)
Distant	177 (24)
Unstaged/unknown	276 (39)
**Treatment**, ***n*** **(%)**	
Radiation only	129 (18)
Surgery only	262 (36)
Surgery+ radiation	78 (11)
Chemotherapy	362 (50)

This table contains the description of demographic and clinical characteristics of 720 PMCTs in the SEER database.

**Table 2:** Racial differences in characteristics of Primary Malignant Cardiac Tumors (PMCTs).

**Characteristics**	**White (*n* = 572)**	**Black (*n* = 75)**	**Asian/ pacific islander (*n* = 73)**	***p*-value**
Age at diagnosis, y ± SD	48 ± 20	39 ± 18	48 ± 20	<0.001
Female *n* (%)	265 (47)	34 (45)	38 (52)	0.630
Hispanics, *n* (%)	113 (20)	1 (1)	2 (3)	<0.001
Insured, *n* (%)	259 (45)	36 (48)	32 (44)	0.869
**Histology** ***n*** **(%)**				<0.001
Angiosarcoma	144 (25)	40 (53)	24 (33)	
Non-angiogenic sarcoma	220 (38)	13 (17)	20 (27)	
Lymphoma	168 (29)	15 (20)	25 (34)	
Mesothelioma	40 (7)	7 (9)	4 (5)	
**SEER staging** ***n*** **(%)**				0.044
Localized	114 (20)	11 (15)	11 (15)	
Regional	92 (16)	20 (27)	17 (23)	
Distant	137 (24)	26 (35)	14 (19)	
Unstaged/unknown	229 (40)	23 (31)	24 (33)	
Treatment, *n* (%)				
Radiation only	102 (19)	11 (15)	17 (23)	0.217
Surgery only	203 (35)	26 (35)	33 (45)	0.153
Surgery+ radiation	61 (11)	6 (8)	11 (15)	0.402
Chemotherapy	284 (50)	40 (53)	38 (52)	0.803

Differences in demographic and clinical characteristics of PMCTs among major races in the United States which include White, Black, and Asian/Pacific Islander races.

### Cancer-specific survival across races

**Figure 1:**
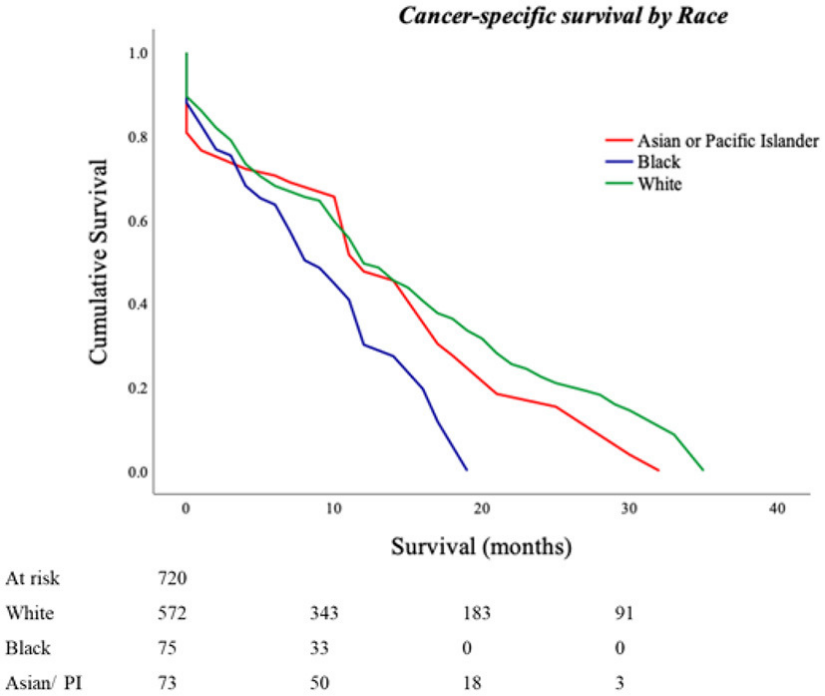
Cancer-specific survival outcomes for Primary Malignant Cardiac Tumors (PMCTs). Black patients have lower cancer-specific survival as compared to White and Asian/Pacific Islander patients in the United States as seen in the SEER database from years 1975 to 2015 (*p*-values; Black vs. White <0.001; Black vs. Asian/Pacific Islander = 0.017, White vs. Asian/Pacific Islander = 0.300).

Six hundred and eighty-four (95%) patients had follow-up data available for a median cancer-specific survival time of 14 (13–15) months. Cancer-specific death rates were 44% for Whites, 32% for Blacks, and 33% for Asians/Pacific Islanders (*p* = 0.040). Kaplan–Meier analysis based on race revealed an estimated cancer-specific survival time of 9.2 ± 1.6 months in Blacks, 13.4 ± 2.6 months in Asian/Pacific Islanders, and 14.8 ± 1.1 months in Whites with a statistically significant pairwise comparison between Blacks vs. Whites and Asians/Pacific Islanders (*p-*values; Black vs. White <0.001; Black vs. Asian/Pacific Islander = 0.017, White vs. Asian/Pacific Islander = 0.300; Figure 1).

### Cancer-specific survival within races (based on histologic type)

#### White

**Figure 2:**
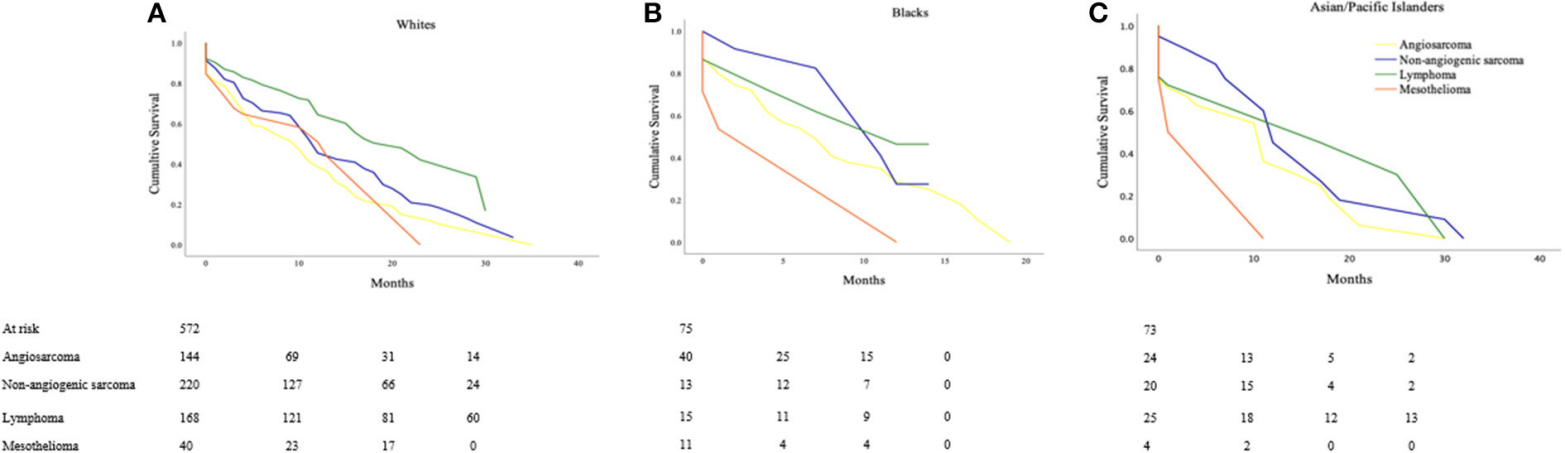
Cancer-specific survival outcomes based on histologic types of Primary Malignant Cardiac Tumors (PMCTs) for various races, including angiosarcomas, non-angiogenic sarcomas, lymphomas and mesotheliomas for **(A)** White (Lymphoma vs. non-angiogenic sarcoma; *p* < 0.001; vs. angiosarcoma; *p* < 0.001; vs. mesothelioma; *p* = 0.046), **(B)** Black (Non-angiogenic sarcoma vs. lymphoma; *p* = 0.956; vs. angiosarcoma; *p* = 0.272; vs. mesothelioma; *p* = 0.296), and **(C)** Asian/Pacific Islanders (Lymphoma vs. non-angiogenic sarcomas; *p* = 0.754; vs. angiosarcoma; *p* = 0.323; vs. mesothelioma; *p* = 0.296).

The estimated cancer-specific survival time was 18.6 ± 2.1 months for lymphomas, significantly higher as compared to 14.0 ± 1.6 months in non-angiogenic sarcomas (*p* <0.001) and 11.5 ± 12.0 months in angiosarcomas (*p* < 0.001). Survival time was lowest at 12.9 ± 3.4 months for mesotheliomas (Lymphoma vs. non-angiogenic sarcoma; *p* < 0.001; vs. angiosarcoma; *p* < 0.001; vs. mesothelioma; *p* = 0.046; Figure 2).

#### Black

In the Black population, estimated cancer-specific survival was the highest in non-angiogenic sarcomas (10.3 ± 2.0 months) followed by lymphomas (9.5 ± 2.9 months), angiosarcomas (8.3 ± 2.1 months), and mesotheliomas (6.6 ± 5.3 months) (Non-angiogenic sarcoma vs. lymphoma; *p* = 0.956; vs. angiosarcoma; *p* = 0.272; vs. mesothelioma; *p* = 0.296; Figure 2).

#### Asian/Pacific Islander

Patients with lymphomas had the highest estimated cancer-specific survival of 17.4 ± 5.4 months followed by non-angiogenic sarcomas at 14.9 ± 5.0 months, angiosarcomas at 10.5 ± 3.7 months, and mesotheliomas at 5.8 ± 5.6 months (Lymphoma; vs. non-angiogenic sarcomas *p* = 0.754; vs. angiosarcoma *p* = 0.323; vs. mesothelioma *p* = 0.296; Figure 2).

### Comparison of cancer-specific survival based on ethnicity for Hispanics and non-Hispanics

**Figure 3:**
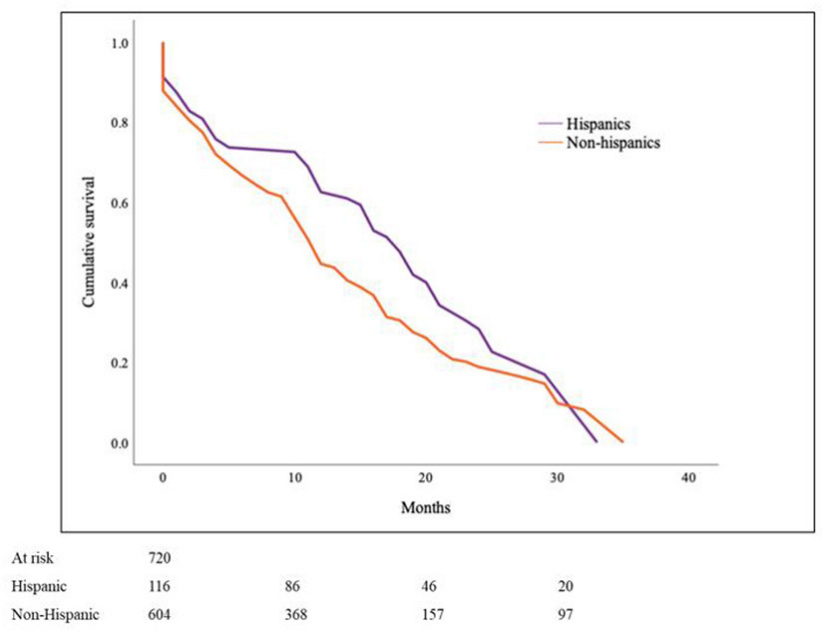
Cancer-specific survival in Primary Malignant Cardiac Tumors (PMCTs) for Hispanics vs. non-Hispanics. Hispanics have a significantly higher survival as compared to non-Hispanics for PMCTs in the SEER database (16.9 ± 2.4 months vs. 13.6 ± 1.1; *p* = 0.011).

One hundred and sixteen (16%) patients in the PMCT cohort were Hispanics. There were 40% females in the Hispanic as compared to 48% in the non-Hispanic group (*p* = 0.090) with the mean age at diagnosis of 40.4 ± 20 years and 48.7 ± 20 respectively (*p* < 0.001). Non-angiogenic sarcomas were more prevalent in Hispanics than non-Hispanics (45 vs. 33%, *p* = 0.017; Table 3). Estimated cancer-specific survival was noted to be 16.9 ± 2.4 months in Hispanics and 13.6 ± 1.1 in non-Hispanics (*p* = 0.011; Figure 3).

**Table 3:** Characteristics of Hispanics with Primary Malignant Cardiac Tumors (PMCTs).

**Characteristics**	**Hispanics (*n* = 116)**	**Non-hispanics (*n* = 604)**	***p*-value**
Age at diagnosis, y ± SD	40.4 ± 20	48.7 ± 20	<0.001
Female, *n* (%)	46 (40)	291(48)	0.092
Insured, *n* (%)	57 (49)	270 (45)	0.379
**Histology**, ***n*** **(%)**			
Angiosarcoma	26 (22)	291 (49)	0.093
Non-angiogenic sarcoma	52 (45)	182 (30)	0.017
Lymphoma	31 (26)	202 (36)	0.574
Mesothelioma	7 (6)	177 (29)	0.631
**SEER staging**, ***n*** **(%)**			
Localized	36 (31)	149 (25)	0.136
Regional	30 (26)	135 (22)	0.383
Distant	29 (25)	155 (26)	0.920
Unstaged/unknown	21 (18)	165 (27)	0.038
**Treatment**, ***n*** **(%)**			
Radiation only	23 (20)	116 (19)	0.876
Surgery only	49 (42)	213 (35)	0.125
Surgery+ radiation	16 (14)	62 (10)	0.262
Chemotherapy	53 (46)	309 (51)	0.281

Table depicting demographic and clinical characteristics of PMCTs in Hispanics and their comparison with non-Hispanic patients.

## Discussion

Population-based registries have been previously used to determine predictors of poor prognosis in PMCTs which include advancing age, comorbidity index, angiosarcoma histology, metastatic disease, chemotherapy, and residual disease (R2) after resection however, the role of race has not been fully understood (10). A relative increase in the utilization of population-based databases to study sociodemographic features of PMCTs has been seen in recent years (11, 12). A study conducted by Heaton et al. on the National Inpatient Sample showed higher in-hospital mortality in Hispanic patients as compared to Whites in a multivariable analysis (*p* = 0.03) (13). Oliveira et al. compared incidences of cardiac and non-cardiac tumors in White, Black, and “Other” races in SEER and found a significantly higher incidence of mesotheliomas of cardiac origin in the Black population (15.9 vs. 4.9%) (1). Saad et al. described a higher incidence of non-angiogenic sarcomas in Whites and angiogenic sarcomas in Blacks in a descriptive manner (14). Bui et al. and Sheth et al. found no differences in overall survival amongst White, Black, and “Other” races in their analyses (15, 16). A limitation of these studies is the representation of various races and tumor histologies as a single category causing an unclear understanding of distinct behaviors in each of the diverse groups included in this disease (17–20). This study effectively explored the SEER database to analyze the role of race and ethnicity on the characteristics and cancer-specific survival outcomes in patients reported to have PMCTs in the United States. The cohort consisted predominantly of White patients, consistent with previous analysis. An interesting finding was the overrepresentation of Asian/Pacific Islanders, almost similar to Blacks suggesting a higher overall prevalence of PMCTs in this race in the United States. However, true prevalence among races may vary due to differential reporting as demonstrated previously in registry-based studies on other cancers (21–23). Alaskan natives continue to be the most underrepresented race with only 6 patients, consistent with prior enrollment data in clinical trials (24). Both sexes were equally represented across races. Black patients were observed to be much younger at diagnosis with a higher incidence of metastatic disease implicating a possible genetic component or delay in access to healthcare. However, differences were noted in the therapeutic modalities used among races suggesting considerable equity in receiving life-prolonging treatment in Blacks.

The PMCTs could be broadly divided histologically into sarcomas, lymphomas, and mesotheliomas based on the data available in SEER. Primary cardiac sarcomas were observed to be the most-commonly diagnosed PMCT, out of which 44% were angiosarcomas, allowing for analysis as a separate group. More than half of Black patients presented with angiosarcomas, accounting for the younger age of presentation in this histological type. Conversely, lymphomas were noted to be the predominant histologic type in Asian/Pacific Islanders as were non-angiogenic sarcomas in Whites.

The primary outcome measure in this study was chosen to be “cause-specific death”, a parameter which has been previously validated in the SEER database. This outcome was thought to provide an advantage in comparing survival as a function of cancer biology in racial minorities as other-cause mortality can be heavily biased by existing healthcare disparities (25). Overall, cancer-specific survival for all PMCTs in this study (~10 months) approximated the all-cause survival duration established in previous studies suggesting a small number of cancer-unrelated deaths. This study demonstrated a significantly worse cancer-specific survival in the Blacks as compared to Whites and Asian/Pacific Islanders. Moreover, cancer-specific survival was longer for lymphomas in Whites and Asian/Pacific Islanders and non-angiogenic sarcomas in Blacks despite no significant difference in treatment. The significantly better survival for lymphomas in White and Asian/Pacific Islander may be a result of availability of better chemotherapeutic options, variability in genetic and/or epigenetic mechanisms and environmental factors (26).

“Hispanic” is term coined by the U.S Federal Government and includes persons of Cuban, Mexican, Puerto Rican, South and Central American, or other Spanish culture regardless of race and origin. They are known to be vulnerable to chronic diseases and have poorer mortality rates which is intricately related to poorer social determinants of health (SDH) such as low literacy and median household income, poorer access to healthcare and lack of insurance (27). A reclassification and survival analysis was in Hispanics as compared to non-Hispanics in this cohort which showed younger age at presentation and poorer survival in Hispanics. These findings shed light on the significant role of social determinants on survival in PMCTs irrespective of race.

## Limitations

Although this study poses several advantages including a relatively larger cohort size, there are several limitations that need to be acknowledged. The study lacked important granular data on individual patients including quality assessment of pathology reports, anatomical site of the tumor, large number of unstaged patients, specific agents used in treatment, and specific reason for death. All non-angiogenic sarcomas have been analyzed as a separate group in this study. However, significant variability in severity and survival exists in different histologic types in this heterogenous group testing which was beyond the scope of this study. Since the size of Black and Asian/Pacific Islanders cohorts was too small, a multivariable analysis could not be performed. Moreover, a selection and reporting bias is possible and absence of information on cancers diagnosed post-mortem may sizably attenuate true incidence. These limitations highlight the need for multicenter collaboration in a randomized controlled fashion to provide future direction.

## Conclusion

Race is an important risk factor in demographic and clinical presentation and cancer-survival in PMCTs with significant variability across histological profiles. The Black race is associated with more severe diseases with poorer survival outcomes. Non-Hispanics and Blacks with Angiosarcoma have worse cancer-specific survival outcomes.

## Data availability statement

The original contributions presented in the study are included in the article/Supplementary material, further inquiries can be directed to the corresponding author/s.

## Ethics statement

Ethical approval was not provided for this study on human participants because the study was exempted from the Wright Center for Graduate Medical Education IRB. Written informed consent for participation was not required for this study in accordance with the national legislation and the institutional requirements.

## Author contributions

MA and HH: analysis and interpretation of data. SS: conception and design, drafting of the manuscript, and revising it critically for important intellectual content. CR and MB revising the manuscript. RN drafting the manuscript. All authors contributed to the article and approved the submitted version.

## Conflict of interest

The authors declare that the research was conducted in the absence of any commercial or financial relationships that could be construed as a potential conflict of interest.

## Publisher's note

All claims expressed in this article are solely those of the authors and do not necessarily represent those of their affiliated organizations, or those of the publisher, the editors and the reviewers. Any product that may be evaluated in this article, or claim that may be made by its manufacturer, is not guaranteed or endorsed by the publisher.

## Abbreviations

PMCTs: Primary Malignant Cardiac Tumors
NCI: National Cancer Institute
SEER: Surveillance, Epidemiology, and End-result.
