# Supplementary material for: Racial and ethnic characteristics and cancer-specific survival in Primary Malignant Cardiac Tumors
Source: Front Cardiovasc Med. 2022 Aug 25;9:961160. doi: 10.3389/fcvm.2022.961160 (PMC9453391; doi:10.3389/fcvm.2022.961160)
Supplement: Supplementary file 1 [file Data_Sheet_1.pdf]

<u>Supplemental material.</u> Histological subtypes of primary malignant cardiac tumors included in the study from the SEER database.
Supplemental table 1. Histologic subtypes of angiogenic sarcomas

**Angiogenic sarcoma, type**	**n**
Hemangiosarcoma	202
Hemangioendothelioma	5
Hemangiopericytoma	1

Supplemental table 2. Histologic subtypes of non-angiogenic sarcomas

**Non-angiogenic sarcoma, type**	**n**
Sarcoma, NOS	55
Spindle cell sarcoma	21
Giant cell sarcoma	27
Epithelioid sarcoma	1
Undifferentiated sarcoma	18
Fibrosarcoma, NOS	13
Fibromyxosarcoma	16
Infantile fibrosarcoma	1
Malignant solitary fibrous tumor	2
Malignant myofibroblastoma	1
Malignant fibrous histiocytoma	12
Myxosarcoma	9
Myosarcoma	27
Epithelioid leiomyosarcoma	1
Angiomyosarcoma	1
Myosarcoma	4
Myxoid leiomyosarcoma	2
Rhabdomyosarcoma	16
Pleiomorphic rhabdomyosarcoma	1
Mixed rhabdomyosarcoma	1
Embryonal rhabdomyosarcoma	5
Alveolar rhabdomyosarcoma	1

Malignant rhabdoid sarcoma	1
Mesenchymoma	1
Synovial sarcoma	10
Synovial spindle cell sarcoma	4
Synovial biphasic sarcoma	2

Supplemental table 3. Histologic subtypes of lymphomas

**Lymphoma, subtype**	**n**
Hodgkin’s lymphoma, mixed cellularity type	1
Nodular sclerosis	6
Precursor NHL, B-cell	2
Chronic/small cell lymphoma	1
Lymphoplasmacytic lymphoma	4
Diffuse large B-cell lymphoma	123
Primary effusion lymphoma	8
Burkitt’s lymphoma	3
Extranodal marginal zone lymphoma, MALT type	4
Follicular lymphoma	6
Plasmacytoma	2
NHL, B-cell	20
Anaplastic large cell/null cell lymphoma	4
Adult T-cell leukemia/lymphoma	1
NHL, NOS	8
Composite Hodgkin’s and NHL	1
Lymphoid neoplasm, NOS	14

Supplemental table 4. Histologic subtypes of mesotheliomas

**Mesothelioma, type**	**n**
Malignant mesothelioma	29
Fibrous mesothelioma	4
Epithelioid mesothelioma	16
Biphasic mesothelioma	2
